# Construction of a self-cloning system in the unicellular green alga *Pseudochoricystis ellipsoidea*

**DOI:** 10.1186/s13068-015-0277-0

**Published:** 2015-06-30

**Authors:** Yuki Kasai, Kohei Oshima, Fukiko Ikeda, Jun Abe, Yuya Yoshimitsu, Shigeaki Harayama

**Affiliations:** Department of Biological Sciences, Faculty of Science and Engineering, Chuo University, Kasuga 1-13-27, Bunkyo-ku, Tokyo, 112-8551 Japan; Research Laboratories, Denso Corporation, Nisshin, Aichi 470-0111 Japan

**Keywords:** *Pseudochoricystis ellipsoidea*, Green algae, Microalgae, Self-cloning, Uracil auxotroph, Genetic transformation, Molecular breeding

## Abstract

**Background:**

Microalgae have received considerable interest as a source of biofuel production. The unicellular green alga *Pseudochoricystis ellipsoidea* (non-validated scientific name) strain Obi appears to be suitable for large-scale cultivation in outdoor open ponds for biodiesel production because it accumulates lipids to more than 30 % of dry cell weight under nitrogen-depleted conditions. It also grows rapidly under acidic conditions at which most protozoan grazers of microalgae may not be tolerant. The lipid productivity of this alga could be improved using genetic engineering techniques; however, genetically modified organisms are the subject of regulation by specific laws. Therefore, the aim of this study was to develop a self-cloning-based positive selection system for the breeding of *P. ellipsoidea*.

**Results:**

In this study, uracil auxotrophic mutants were isolated after the mutagenesis of *P. ellipsoidea* using either ultraviolet light or a transcription activator-like effector nuclease (TALEN) system. The cDNA of the uridine monophosphate synthase gene (*PeUMPS*) of *P. ellipsoidea* was cloned downstream of the promoter of either a beta-tubulin gene (*PeTUBULIN1*) or the gene for the small subunit of ribulose 1,5-bisphosphate carboxylase/oxygenase (*PeRBCS*) to construct the pUT1 or pUT2 plasmid, respectively. These constructs were introduced into uracil auxotroph strains, and genetically complementary transformants were isolated successfully on minimal agar plates. Use of Noble agar as the solidifying agent was essential to avoid the development of false-positive colonies. It took more than 6 weeks for the formation of colonies of pUT1 transformants, whereas pUT2 transformants formed colonies in 2 weeks. Real-time PCR revealed that there were more *PeUMPS* transcripts in pUT2 transformants than in pUT1 transformants. Uracil synthesis (Ura^+^) transformants were also obtained using a gene cassette consisting solely of *PeUMPS* flanked by the *PeRBCS* promoter and terminator.

**Conclusions:**

A self-cloning-based positive selection system for the genetic transformation of *P. ellipsoidea* was developed. Self-cloned *P. ellipsoidea* strains will require less-stringent containment measures for large-scale outdoor cultivation.

**Electronic supplementary material:**

The online version of this article (doi:10.1186/s13068-015-0277-0) contains supplementary material, which is available to authorized users.

## Background

Microalgae have received considerable interest as a source of biofuel production. In particular, the biodiesel produced by microalgal biomass may be a viable alternative to petroleum-based and land-plant-based fuels because microalgae have several advantages over terrestrial plants for biofuel production, such as low land requirements and high oil yields [1–3]. Although the production of crude oil from algal biomass has already been achieved in various pilot-scale facilities, the current production costs of algal oil are not competitive with those of fossil oil [4]. The processes used to produce crude algal oil include cultivation and recovery of algal biomass, oil extraction from the algal biomass, and conversion of fatty acid moieties of the oil to liquid biofuels using either methyl esterification or hydrogenation. Currently, efforts are underway to reduce the capital and operating costs of these processes [5, 6]. However, significant cost reduction could also be achieved via algae breeding, particularly using genetic engineering techniques [7, 8].

Genetically modified algal strains are subject to laws concerning biosafety and also raise ambiguous public concerns; therefore, molecular breeding using techniques based on self-cloning and/or *natural occurrence* (cloning DNA from a donor into a recipient, between which the natural exchange of DNA is possible) was proposed as a desirable method to construct recombinant organisms for deliberate release into the environment. In Japan, recombinant microorganisms including microalgae, obtained using self-cloning or natural occurrence are exempt from the restrictions imposed by the Cartagena domestic law [9, 10].

In plants, concepts named *cisgenesis* and *intragenesis*, which are analogous to those of self-cloning and natural occurrence, have been proposed. In cisgenesis and intragenesis, fragments of non-recombinant and recombinant DNA, respectively, from a sexually compatible donor organism is introduced into a recipient. Since both cases involve the exchange of DNA between interbreeding groups, the regulatory measures imposed on cisgenesis and intragenesis could be similar to those for conventional breeding. The regulation of intragenic and cisgenic organisms is currently under discussion in many countries, including in the European Union and the United States, and the regulation of these organisms might be relaxed soon [11]. Amid such trends, the development of self-cloning technology is important for algae breeding using outdoor open pond cultivation, which is currently considered the most viable option for the large-scale cultivation of microalgae [12].

*Pseudochoricystis ellipsoidea* (non-validated scientific name) strain Obi is a unicellular green alga classified within the family of *Trebouxiaceae*, which accumulates lipids to an amount of more than 30 % of the dry cell weight under nitrogen-depleted conditions [13]. During nitrogen depletion, the amount of neutral lipids increases, whereas chloroplast-specific glycolipids decrease [14]. A peculiar characteristic of this alga is its ability to grow rapidly under acidic conditions below pH 3.5 without reducing the lipid productivity (Kurano et al., in preparation). Since most protozoan grazers of microalgae might not tolerate that pH, *P. ellipsoidea* might be suitable for large-scale cultivation in outdoor open ponds for biodiesel production. Thus, we developed a method for the genetic transformation of this alga [15] and are using this method to genetically improve the oil productivity of *P. ellipsoidea*.

We were also interested in developing a self-cloning-based positive selection system for the breeding of *P. ellipsoidea* for two reasons. First, as discussed above, self-cloning is subject to less-stringent regulation [16, 17], and a consensus regarding the deregulation of the deliberate release of self-cloned microalgae is likely to be built among international experts in the near future. If this occurs, a climate could be developed to foster the cultivation of self-cloned microalgae in outdoor open ponds. Second, genetic engineering includes the controlled overexpression and the targeted inactivation of genes, which both require the use of selectable marker genes to acquire desired clones. Since only one marker is currently available in *P. ellipsoidea* (G418 resistance), auxotrophic markers will serve as additional selectable markers.

In the current study, we report (i) the isolation of uracil-requiring mutants of *P. ellipsoidea* that are defective in uridine monophosphate synthetase (UMPS), (ii) the synthesis of the full-length cDNA for UMPS, and (iii) the complementation of *P. ellipsoidea* uracil auxotrophs by introducing UMPS cDNA. We encountered several problems that were resolved. This information will be useful for individuals interested in constructing self-cloning systems in other microalgae.

## Results and discussion

### Isolation of uracil auxotrophs

During the ultraviolet light (UV)-mediated mutagenesis of *P. ellipsoidea* cells, the survival of UV-irradiated cells decreased over UV-C irradiation time and reached 0.1 % after 4 min (see Additional file 1). The mutated cells were screened for the uracil-requiring (Ura^−^) phenotype on MA5 minimal agar plates containing both 5-fluoroorotic acid (5-FOA) and uracil. Thirty-six 5-FOA-resistant colonies were isolated after a UV-C dose yielded a survival rate of ~50 %, and five of these were uracil auxotrophs. Using genomic DNAs isolated from two of the five uracil auxotrophs named strains M1 and M2, DNA fragments encompassing the coding region (CDR) of the gene for UMPS (*PeUMPS*) were PCR-amplified, and sequenced. Eukaryotic UMPS is a bifunctional enzyme that consists of orotate phosphoribosyltransferase (OPRTase) and orotidine-5′-phosphate decarboxylase (ODCase), which catalyze the last two steps of the *de novo* UMP biosynthetic pathway [18]. The DNA sequencing analysis showed that a 5-base insertion and a 27-base deletion were present in *PeUMPS* in strains M1 and M2, respectively, both in the OPRTase domain. The spontaneous reversion frequency to Ura^+^ was 7.1 × 10^−6^ for strain M1, and less than 10^−7^ for strain M2. Because of the instability of the M1 mutation, strain M1 was not characterized further. Whole-genome sequencing of strain M2 covering 99.4 % of the whole genomic sequence of *P. ellipsoidea* strain Obi (Harayama et al*.*, in preparation) revealed the presence of a one-base substitution in an intron of a gene of unknown function in addition to the *PeUMPS* mutation described above.

Two uracil auxotrophs named strains M3 and M4 were also isolated from a *P. ellipsoidea* strain Obi derivative carrying two DNA fragments encoding transcription activator-like effector nuclease (TALEN) right and left arms that were designed for the mutagenesis of *PeUMPS*. The M3 and M4 strains were identified after selecting 5-FOA-resistant colonies grown on MA5 minimal agar plates containing uracil and 5-FOA. The M3 and M4 strains had a 29-base insertion and a 192-base deletion, respectively, in the ODCase domain of *PeUMPS*. However, these mutations were outside the TALEN-target sequence, suggesting that they arose independently from TALEN activity. The frequency of reversion of the two mutants to Ura^+^ was less than 10^−7^.

The M3 and M4 strains that carry the TALEN right and left sequences would not be appropriate hosts for self-cloning. Nevertheless, we used these strains together with strain M2 in following complementation analyses because the mutation loci in the M3 and M4 strains were within the OPRTase domain which was different from the mutation locus within the ODCase domain in strain M2.

### Cloning and functional analysis of *PeUMPS* cDNA

**Fig. 1:**
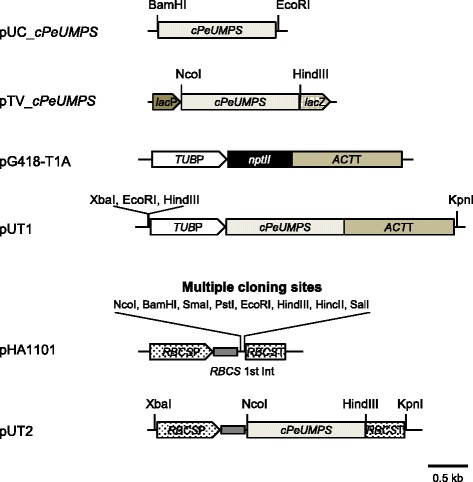
Construction of the plasmids used in this study. The genes and loci are abbreviated as follows: *cPeUMPS PeUMPS* cDNA; *lacP*, the promoter and operator regions of the *E. coli* beta-galactosidase gene (*lacZ*); *nptII*, the gene for neomycin phosphotransferase II to confer G418 resistance; *TUB*P, the promoter region of *PeTUBULIN1*; *ACT*T, the terminator region of *PeACT1*; *RBCS*P, the promoter region of *PeRBCS*; *RBCS*T, the terminator region of *PeRBCS*; *RBCS* 1st Int, the first intron of *RBCS*

A 1.5-kb *PeUMPS* cDNA was cloned into pUC119 to construct pUC_c*PeUMPS* (Fig. 1). The cloned *PeUMPS* cDNA sequence was 100 % identical to that predicted from the genomic sequence of *P. ellipsoidea* strain Obi. The pTV-c*PeUMPS* plasmid (Fig. 1) was constructed to express *PeUMPS* cDNA in two uracil auxotrophic mutants of *Escherichia coli*: JD24489 (an OPRTase mutant) and JW1273 (an ODCase mutant). Both *E. coli* mutants did not grow on glucose M9 minimal medium, whereas clones that possessed pTV-c*PeUMPS* grew efficiently, suggesting that the *PeUMPS* cDNA was expressed functionally in *E. coli*.

### Genetic complementation of uracil auxotrophic *P. ellipsoidea* mutants using *PeUMPS* cDNA

Plasmid pUT1 containing *PeUMPS* cDNA flanked by the promoter of *PeTUBULIN1* and the terminator of an actin gene (*PeACTIN1*) (Fig. 1) was introduced into the uracil auxotrophic strain M4, and prototrophic transformants were selected on MA5 minimal medium solidified using Bacto agar (BD Difco). However, prototrophic transformants could not be selected because of the background growth of strain M4 on MA5 minimal agar plates even when M4 cells grown in MA5 medium supplemented with uracil were washed extensively before plating (data not shown). To reduce the background cell growth, both pG418-T1A [15] (Fig. 1) and pUT1 were co-introduced into strain M4, and the resulting G418-resistant transformants were screened on MA5 minimal agar plates containing G418 and uracil.

**Fig. 2:**
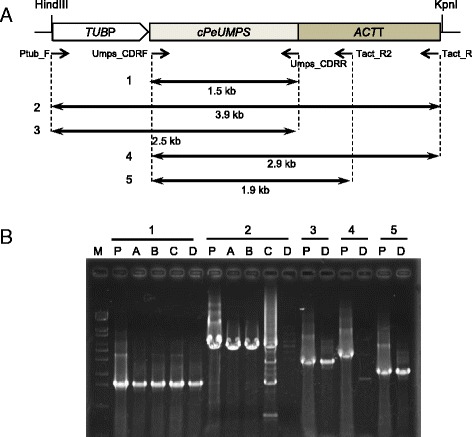
PCR amplification of the pUT1 sequences integrated into the genomes of transgenic strains. **a** The pUT1 map: *TUB*P, the promoter region of *PeTUBULIN1*; *cPeUMPS*, the *PeUMPS* cDNA; *ACT*T, the terminator region of *PeACT1*. The positions of the five PCR primers and the PCR products amplified using the primers (1–5) are shown below the pUT1 map. **b** Agarose gel electrophoresis to verify the presence or absence of PCR products 1–5. *M*, DNA marker (λ-EcoT14 I digest); *P*, pUT1; *A*, 4 M-1A; *B*, 4 M-1B; *C*, 4 M-1C; *D*, 4 M-1D

Six G418-resistant colonies were obtained, and the existence of pUT1 sequences in the transformants grown on MA5 minimal agar plates containing uracil was examined using PCR with two forward primers (one that hybridized with the 5′-ends of the *PeTUBULIN1* promoter, and another that hybridized with the 5′-ends of the *PeUMPS* cDNA) and three reverse primers that hybridized the 3′-end of the *PeUMPS* cDNA, the middle part of the *PeACTIN1* terminator, and the 3′-end of the *PeACTIN1* terminator (Fig. 2). In the genomes of three out of the six transformants (named strains M4-1A, M4-1B, and M4-1C), the DNA region covering the *PeTUBULIN1* promoter, *PeUMPS* cDNA, and the *PeACTIN1* terminator was integrated. Conversely, the genome of the transformant named strain M4-1D contained a region covering the *PeTUBULIN1* promoter, *PeUMPS* cDNA, and a 0.4-kb 5′-end of the *PeACTIN1* terminator (Fig. 2). The PCR products amplified from the M4-1C genome using the primers Ptub_F and Tact_R were a mixture of four or more DNA fragments (Fig. 2b, lane 2_C), suggesting that the DNA region, the *PeTUBULIN1* promoter–*PeUMPS* cDNA–the *PeACTIN1* terminator, integrated in the genome of the transgenic M4-1C strain was unstable when grown in the presence of uracil.

When these four *PeUMPS*-cDNA-positive transgenic strains were streaked onto uracil-free MA5 agar plates, colonies developed only after incubation for 6–8 weeks at 25 °C. Since strain M4 formed no colony on uracil-free MA5 agar plates after 8 weeks, the introduced *PeUMPS* cDNA complemented the *PeUMPS* mutation in strain M4. But the effect was very weak. To shorten the time required for colony formation, MA5 minimal agar plates supplemented with precursors of the UMP biosynthetic pathway (L-glutamate, orotate, and phosphoribosyl pyrophosphate) were prepared, and the growth of the four transgenic strains on the plates was examined. The transgenic strains M4-1C and M4-1D formed colonies in 3 weeks on MA5 minimal agar plates containing orotate; however, the growth improvement by orotate was not significant in the M4-1A and M4-1B strains, which still formed colonies in only 8 weeks. Neither phosphoribosyl pyrophosphate nor L-glutamate improved the growth of any transgenic strains.

**Fig. 3:**
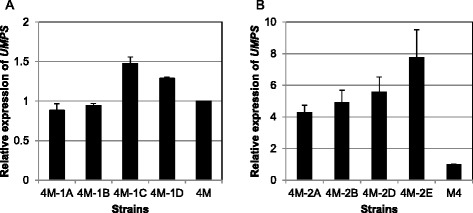
Real-time PCR analysis of the expression of *PeUMPS* in (**a)** pUT1 and (**b)** pUT2 transgenic strains. 18S rRNA was used as an internal control. Strain M4 is the parental strain of the transgenic strains. Real-time PCR detected expression from the defective *PeUMPS* in strain M4 in addition to the transgenic *PeUMPS* cDNA. The data are presented as fold changes in *PeUMPS* expression normalized to 18S rRNA expression in transgenic strains compared with strain M4. *Bars* represent the standard deviation of three replicates

To evaluate the correlation between the expression of *PeUMPS* cDNA and the growth speed, the expression of *PeUMPS* cDNA in the four transgenic strains was analyzed using real-time PCR and normalized against 18S rRNA levels (Fig. 3a). The PCR primers Umps_QRT_F and Umps_QRT_R were used to detect both the expression of transgenic *PeUMPS* cDNA and the expression of defective *PeUMPS* in the chromosome of strain M4. Strain M4-1C, which grew fastest (its colonies were visible in 6 weeks), exhibited a 1.5-fold higher expression than did strain M4. It suggested that the expression of the transgenic *PeUMPS* cDNA in strain M4-1C was 1.5−1-fold of the expression of the original *PeUMPS*. Similarly, the expression of the transgenic *PeUMPS* cDNA in strain M4-1D was approximately one quarter of the expression of the original *PeUMPS*. Strain M4-1D grew faster than strains M4-1A and M4-1B in the presence of orotate. In contrast, no transgene expression was detectable in strains M4-1A and M4-1B, which grew very slowly even in the presence of orotate. These results suggest that the slow growth of the transgenic strains on MA5 minimal agar plates was due to the low expression of the transgenic *PeUMPS* cDNA.

**Fig. 4:**
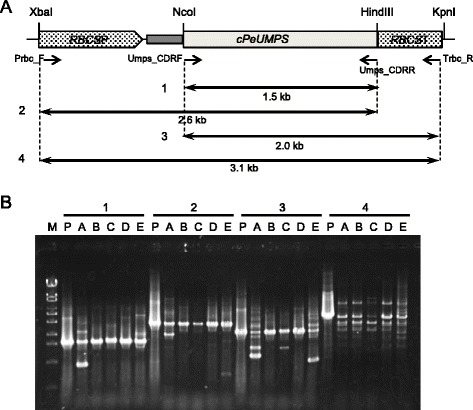
PCR amplification of the pUT2 sequence that had integrated into the genomes of transgenic strains. **a** The pUT2 map: *RBCS*P, the promoter region of *PeRBCS*; *cPeUMPS*, *PeUMPS* cDNA; *RBCS*T, the terminator region of *PeRBCS*. The positions of the four PCR primers and PCR products amplified using the primers (1–4) are shown below the pUT2 map. **b** Agarose gel electrophoresis to verify the presence or absence of PCR products 1–4. M, DNA marker (λ-EcoT14 I digest); P, pUT2; A, 4 M-2A; B, 4 M-2B; C, 4 M-2C; D, 4 M-2D; E, 4 M-2E

We next attempted to place the *PeUMPS* cDNA under the control of a stronger promoter. Whole transcriptome shotgun sequencing (RNA-seq) was applied to *P. ellipsoidea* strain Obi, which revealed that mRNA of the gene for ribulose-1,5-bisphosphate carboxylase/oxygenase small subunit (*PeRBCS*) was the highest among all nuclear genes and was almost 20 times higher than was that of *PeTUBLIN1* (Ide et al., unpublished data). Thus, the *PeUMPS* cDNA fragment was inserted downstream of the *PeRBCS* first intron in plasmid pHA1101 to construct plasmid pUT2 (Fig. 1). The pUT2 and pG418-T1A plasmids were then used to co-transform strain M4 using particle bombardment. Among the 106 G418-resistant colonies that developed, the *PeUMPS* cDNA sequence was identified in eight colonies using PCR. To determine the pUT2 sequences that integrated in the genome of these eight transgenic strains, PCR was performed using three primer sets to detect the 5′-end of the *PeRBCS* promoter, the middle part of the *PeUMPS* cDNA, and the 3′-end of the *PeRBCS* terminator, respectively (Fig. 4). All three regions were detected in five transgenic strains (M4-2A, M4-2B, M4-2C, M4-2D, and M4-2E), whereas the remaining three transgenic strains contained the *PeUMPS* cDNA and *PeRBCS* terminator regions, but not the *PeRBCS* promoter region (data not shown). The PCR products amplified from the genome of strain M4-2A contained multiple fragments, suggesting that a population of strain M4-2A harbored partial deletions in the integrated expression cassette of the *PeRBCS* promoter–*PeUMPS* cDNA–the *PeRBCS* terminator. Thus, the expression cassette in strain M4-2A was unstable to a certain extent in strain M4-2A. Using genomic DNA isolated from strain M4-2C, fragments 2 and 4 were not amplified efficiently. We interpreted this result as suggesting that strain M4-2C did not carry the full-length expression cassette, but instead carries two or more incomplete expression cassettes: at least one covering the *PeRBCS* promoter—*PeUMPS* cDNA region, and one covering the 3′-end *PeUMPS* cDNA—the *PeRBCS* terminator region.

Strains M4-2A, M4-2B, M4-2D, and M4-2E grew on MA5 minimal agar plates and formed colonies within 7 days, similar to the wild-type *P. ellipsoidea* strain Obi. However, strain M4-2C grew on MA5 minimal agar plates and formed colonies after only 6 weeks. Strain M4-2C grew slower than other transgenic strains probably because the strain only carried incomplete forms of the gene expression cassette, as described above.

The expression of *PeUMPS* cDNA in strains M4-2A, M4-2B, M4-2D, and M4-2E were determined using real-time PCR and normalized to 18S rRNA (Fig. 3b). The expression of the transgene was three- to seven-fold higher than was the expression of the chromosomal copy of *PeUMPS*.

Southern blotting of genomic DNAs extracted from strains M4-1A, M4-1B, M4-1C, M4-1D, M4-2A, M4-2B, M4-2D, and M4-2E was performed, and the results are shown in Additional file 2. Southern blotting analysis of genomic DNAs digested with EcoRI should yield two DNA fragments of 8 and 3 kb (see Additional file 2) originated from the original *UMPS* gene, that were hybridized to the *UMPS* cDNA probe. These two bands were detected in all DNA samples, including that from strain M4. In each of the transgenic strains, additional band(s) were detected, and the number of the additional bands indicated the copy number of *UMPS* cDNAs that integrated into the genome. Thus, the copy numbers were expected to be 2, 4, 4, and 4 for strains M4-1A, M4-1B, M4-1C, and M4-1D, respectively, and 7, 1, 4, and 7 for strains M4-1A, M4-1B, M4-1D, and M4-1E, respectively, based on the Southern blots obtained using EcoRI-digested genomic DNA. Similar results were obtained from Southern blotting performed using XbaI-digested genomic DNA (see Additional file 2). Since strain M4-2B, which carried a single copy of *PeUMPS* cDNA under the control of the *PeRBCS* promoter, expressed the transgene at a much higher than did the transgenic strains carrying multiple copies of *PeUMPS* cDNA under the control of the *PeTUBULIN1* promoter, we concluded that the transgene was expressed at significantly higher levels from the *PeRBCS* promoter compared with the *PeTUBULIN1* promoter.

**Fig. 5:**
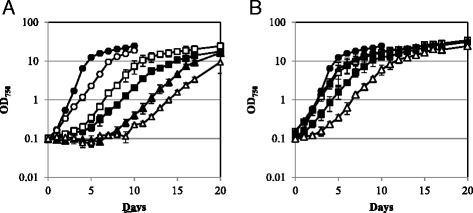
Growth of transgenic strains in MA5 minimal medium. **a** *P. ellipsoidea* strain Obi (*closed circles*), M4 in the presence of 1 mM uracil (*open circles*), M4-1A (*closed squares*), M4-1B (*open squares*), M4-1C (*closed triangles*), and M4-1D (*open triangles*). **b** *P. ellipsoidea* strain Obi (*closed circles*), M4-2A (*closed squares*), M4-2B (*open squares*), M4-2D (*closed triangles*), and M4-2E (*open triangles*). Experiments were performed in triplicate; *bars* represent the standard deviation

Analysis of the growth of the transgenic strains in MA5 minimal liquid medium showed that transgenic strains containing the pUT1 sequence started to grow with a lag phase of 2–8 days and slower growth rates compared with the wild-type strain (*P. ellipsoidea* strain Obi) and strain M4 in the presence of 1 mM uracil (Fig. 5a). In contrast, two of the transgenic strains carrying the pUT2 sequence (4 M-2B and 4 M-2D) grew in MA5 minimal liquid culture at growth rates equivalent to wild-type *P. ellipsoidea* strain Obi, whereas the other two transgenic strains (4 M-2A and 4 M-2E) grew slower than the wild-type strain, despite high levels of transgene expression (Fig. 5b). Although the reason for the slower growth of strains 4 M-2A and 4 M-2E is unclear, it is possible that the insertion of a copy of *PeUMPS* cDNA or the G428-resistance gene into the genomes of these strains disrupted gene(s) that are essential to the rapid growth of *P. ellipsoidea*.

In the studies described above, the *PeUMPS* cDNA was introduced into strain M4 using a co-transformation technique with pG418-T1A plasmid to first select G418-resistant transformants. However, since we demonstrated the practical utility of pUT2 plasmid for complementing the defect in *PeUMPS*, we next attempted to select Ura^+^ transformants of the uracil auxotroph strains M2, M3, and M4 using this plasmid. As described above, the background growth of these auxotrophs on MA5 minimal agar plates hampered the effective selection of Ura^+^ transformants. Nevertheless, we found that the use of Noble agar (BD Difco) instead of Bacto agar eliminated background growth of the uracil auxotrophic strains on MA5 minimal agar plates completely. Thus, plasmid pUT2 was introduced in strains M2, M3, and M4, and Ura^+^ transformants were selected directly on MA5 minimal agar plates solidified using Noble agar (BD Difco). Colonies of Ura^+^ transformants of strains M2, M3, and M4 appeared in 10 days, whereas no colony appeared on the agar plates containing bombarded microcarriers without DNA coating. The transformation rates differed among the uracil auxotrophic strains (Table 1); that of strain M4 was significantly lower than were those of strains M2 and M3.

**Table 1:** Transformation efficiencies

Strain	Number of transformants/μg DNA	
	pG418-T1A	pUT2
M2	42.0 ± 13.8	122.5 ± 15.0
M3	19.1 ± 5.1	123.5 ± 4.3
M4	18.0 ± 3.2	15.7 ± 1.7

To establish a method for genetic transformation without introducing foreign nucleic acid sequences (self-cloning), a gene cassette consisting of the *PeRBCS* promoter–*PeUMPS* cDNA–the *PeRBCS* terminator was excised from the pUT2 plasmid using restriction-enzyme digestion, and purified after gel electrophoresis. The linearized gene cassette DNA was then introduced into strain M2 using particle bombardment, and Ura^+^ transformants were selected on MA5. Ura^+^ colonies appeared in 10 days at a frequency of 57.3 ± 6.9 transformants/μg DNA. Although this value was half the transformation frequency obtained using pUT2, it was sufficient for the practical use of the gene cassette for the genetic transformation of Ura^−^ strains of *P. ellipsoidea*.

## Conclusions

In this study, a self-cloning-based positive selection system was developed in three distinct steps to obtain transgenic derivatives of *P. ellipsoidea* strain Obi. In the first step, four uracil auxotrophic mutants named strains M1 to M4 were isolated by selecting 5-FOA-resistant derivatives. Strain M2, which was isolated after weak UV mutagenesis, was most convenient as a recipient in genetic transformation because it contained only two mutations: a 27-base deletion in *PeUMPS*, and a base substitution in an intron of a gene of unknown function. In the second step, UMPS cDNA was cloned and sandwiched between a promoter sequence and a terminator sequence. The *PeRBCS* promoter was suitable for the transgenic expression of *PeUMPS*, whereas the *PeTUBULIN1* promoter did not support sufficient transgenic expression of *PeUMPS.* In the third step, the preparation of minimal agar plates using Noble agar was critical for the efficient screening of Ura^+^ transformants. The information obtained in this study will be useful for researchers interested in constructing self-cloning systems in other microalgae.

## Methods

### Algal strain and culture conditions

The algal and bacterial strains used in this study are listed in Table 2. *P. ellipsoidea* strain Obi (wild-type) and its uracil auxotrophic mutants were cultured in a 120-ml test tube containing 50 ml of MA5 medium [15] or MA5 medium supplemented with 1 mM uracil under continuous illumination with daylight fluorescent tubes (40 W FL40S・FR・P, Panasonic) at 100 μmol photons m^−2^ s^−1^ in a plant-growth chamber (type #CLE-303, Tomy). The media were bubbled with 1 % (*v*/*v*) CO_2_ at 25 °C. For agar-plate cultivation, the media were solidified using 1.5 % agar (Bacto agar or Noble agar, BD Difco), and the resulting agar plates were incubated in the plant-growth chamber.

**Table 2:** List of *P. ellipsoidea* and *E. coli* strains used in this study

Strains	Genotype	Source or reference
*P. ellipsoidea*		
Obi	Wild-type	[13]
M2	Carry a 27-base deletion within the OPRTase domain of UMPS	This study
M3	Carry a 29-base insertion within the ODCase domain of UMPS	This study
M4	Carry a 192-base deletion within the ODCase domain of UMPS	This study
TAL-1	Carry two chromosomally integrated DNA fragments encoding TALEN left and right arms	This study
*E. coli*		
DH5α	F^−^ *endA1*, *glnV44*, *thi-1*, *recA1*, *relA1*, *gyrA96*, *deoR*, *nupG*, Φ80d*lacZ*ΔM15, Δ(*lacZYA-argF*)U169, *hsdR17* (rK^−^ mK^+^), λ^−^	TOYOBO
JD24489	W3110 (typeA) *lacIq*, *lacZ*ΔM15, *galK*2, *galK*22, ∆*pyrE*::kan	National Institute of Genetics
JW1273	∆(*araD*-*araB*)*567*, ∆*lacZ4787*(::*rrnB-3*), λ^−^, ∆*pyrF789*::kan, *rph-1*, ∆ *rhaD*-*rhaB*)*568*, *hsdR514* (rK^−^ mK^+^)	National Institute of Genetics

### Isolation of uracil auxotroph mutants

Two methods were used to isolate uracil-requiring mutants: UV mutagenesis and TALEN mutagenesis. For UV mutagenesis, *P. ellipsoidea* strain Obi cells cultured at an OD_750_ of 2.2 were collected by centrifugation at 5000×*g* for 5 min, and then re-suspended in 50 mM potassium phosphate buffer (pH 7.4) to an OD_750_ of 1 (approximately 2 × 10^7^ cells ml^−1^). Two milliliters of the cell suspension was transferred into an empty Petri dish, stirred using a stirring bar, and irradiated with an ultraviolet (UVC) lamp (15 W GL15, Mitsubishi Electric) for 1–15 min at a distance of 6 cm. Subsequently, the irradiated cells were stored in the dark for 18 h at 4 °C to prevent photoreactivation, and then each cell suspension was added to 25 ml of MA5 medium containing 1 mM Na_2_CO_3_ and 1 mM uracil and incubated photosynthetically in the plant-growth chamber until it reached an OD_750_ of more than 5 to segregate mutated genes. Cells in these cultures were diluted serially, and then spread on MA5 minimal agar plates containing 1 mM uracil and 4.3 mM 5-FOA (Wako Pure Chemical Industries). The agar plates were incubated for 2 weeks in the plant-growth chamber, and the colonies that grew on the agar plates were picked using sterile toothpicks and streaked on fresh MA5 minimal agar plates containing 1 mM uracil and 3.9 mM 5-FOA for single colony isolation. Each of single colonies that developed on the agar plates was picked using a toothpick and streaked first on an MA5 agar plate and then on an MA5 minimal agar plate containing 1 mM uracil. Among the uracil auxotroph mutants that grew on an MA5 minimal agar plate containing 1 mM uracil but not on an MA5 minimal agar plate, two strains isolated from a culture that had been exposed to a minimal dose of UV irradiation (approximately 1 min of irradiation) were named strains M1 and M2, and maintained.

Strain TAL-1 is a derivative of *P. ellipsoidea* strain Obi that carries in its genome two DNA fragments (TALEN [19–21] right and left arms) that are designed specifically for the mutagenesis of *PeUMPS*. Two uracil auxotrophs named strains M3 and M4 were isolated from this strain by selecting 5-FOA-resistant colonies on MA5 minimal agar plates containing uracil and 5-FOA.

Genomic DNAs were extracted from these mutants as described by Imamura et al. [15], and the entire *PeUMPS* sequence was PCR-amplified from each DNA sample using the primers Umps_F (5′-CCACAAAACCCATTGCCTCAACA-3′) and Umps_R (5′-TCCGTGCTGTCTTCCAGGTCTT-3′) (Table 3). The *PeUMPS* sequences in the two mutants were determined using dideoxy chain termination via a commercial service provided by Macrogen Japan Corp.

**Table 3:** List of PCR primers used in this study

Primer name	Sequence (5′ to 3′)	Target
Umps_F	CCACAAAACCCATTGCCTCAACA	5′-end of *UMPS*
Umps_R	TCCGTGCTGTCTTCCAGGTCTT	3′-end of *UMPS*
Umps_cDNAF	CCAAGCTTTGCATGGCATTCAACAAGGTGAA	5′-end of *UMPS* cDNA
Umps_cDNAR	GGAATTCCCAACCTCATTTGAGAGGCAAT	3′-end of *UMPS* cDNA
Umps_CDRF	CACCCATGGCAACTTCAACTCCCTCAGT	5′-end of *UMPS* CDR
Umps_CDRR	CACAAGCTTCATGCCAGCGTGGCCTCATA	3′-end of *UMPS* CDR
Ptub_F	CCAAGCTTTGCATGGCATTCAACAAGGTGAA	*TUBULIN1* promoter
Ptub_R_Umps5′	GAGTTGAAGTTGCCATCTTGAACGACTTCCCTCGACAA	5′-end of *UMPS* CDR and 3′-end of *TUBULIN* promoter
Umps3′_Tact_F	GCCACGCTGGCATGAGGTGAGCCTGAAACAGCTTTCTG	3′-end of *UMPS* CDR and 5′-end of *ACT1* terminator
Tact_R	TCGGTACCGGTTGTTGAGTTGAGCACTGGAC	3′-end of *ACT1* terminator
Tact_R2	CCCCAGCTGCTACTGCTATC	A middle part of *ACT1* terminator
Prbc_F	CTCAATCGGCCGATTTCATGCATGA	5′-end of *RBCS* promoter
Trbc_R	GTGCTGGTCTGTTTCCATGCAGTCAT	3′-end of *RBCS* terminator
Ptub3′_Umps_CDRF	GGGAAGTCGTTCAAGATGGCAACTTCAACTCCCTCAG	3′-end of *TUBULIN* promoter and 5′-end of *UMPS* CDR
Umps_CDRR_Tact5′	TGTTTCAGGCTCACCTCATGCCAGCGTGGCCTCATA	5′-end of *ACT1* terminator and 3′-end of *UMPS* CDR
G418F	GATCGGCCATTGAACAAGAT	*nptII*
G418R	GCGATACCGTAAAGCACGAG	*nptII*
Umps_QRT_F	TCTTCGACAAGTGGGATGATGG	Position 908–930 of *UMPS* CDR
Umps_QRT_R	TGTTACCGATGTCCGCAAAC	Position 993–1012 of *UMPS* CDR
18SrRNA_QRT_F	GGATCAATTGGAGGGCAAGT	Position 627–646 of 18S rRNA
18SrRNA_QRT_R	GCCCGAAATCCAACTACGAG	Position 713–732 of 18S rRNA

### Cloning of the cDNA encoding UMPS

Standard molecular procedures were performed as described by Sambrook et al [22]. Total RNA was extracted from *P. ellipsoidea*” strain Obi cells grown to an OD_750_ of 1.2 using an RNeasy Mini kit (Qiagen), and any remaining genomic DNA was digested using an RNase-free DNase Set (Qiagen) according to the manufacturer’s instructions. The RNA concentration was determined from the absorbance at 260 nm, and the RNA integrity was examined using agarose gel electrophoresis. First-strand cDNA was synthesized using a Transcriptor High Fidelity cDNA Synthesis kit (Roche) with anchored-oligo (dT)_18_ primers according to the manufacturer’s instructions. A fragment of *PeUMPS* cDNA was amplified from a cDNA library using PCR with the primers Umps_cDNAF (5′-CGGGATCCCGCTGCATCTTTGCTTTTGTGC-3′; a BamHI site is underlined) and Umps_cDNAR (5′-GGAATTCCCAACCTCATTTGAGAGGCAAT-3′; an EcoRI site is underlined). The resulting amplified fragment was digested using BamHI and EcoRI and ligated into the BamHI and EcoRI sites of pUC119 to create pUC-c*PeUMPS*. The sequence of the resulting *PeUMPS* cDNA was verified using double-stranded sequencing as described above. To construct a *PeUMPS* cDNA expression vector in *E. coli*, PCR was performed using the primers Umps_CDRF (5′-CACCCATGGCAACTTCAACTCCCTCAGT-3′; an NcoI site is underlined) and Umps_CDRR (5′-CACAAGCTTCATGCCAGCGTGGCCTCATA-3′; a HindIII site is underlined). The amplified fragments were then digested using NcoI and HindIII and cloned into the NcoI and HindIII sites of pTV118 (TaKaRa) to create pTV_c*PeUMPS*. To confirm the functionality of the cloned *PeUMPS* cDNA, pTV_c*PeUMPS* was introduced into the uracil auxotrophic *E. coli* strains JD24489 [W3110 (type A), *lacI*^*q*^, *lacZ*ΔM15, *galK*2, *galK*22, ∆*pyrE*::kan] and JW1273 [*∆*(*araD*-*araB*)*567*, *∆lacZ4787*(::*rrnB-3*), λ^−^, *∆pyrF789*::kan, *rph-1*, *∆*(*rhaD*-*rhaB*)*568*, *hsdR514*] [23], which were purchased from the National Institute of Genetics of Japan (Mishima). The growth of the *E. coli* transformants was examined on glucose M9 minimal medium [22].

### Construction of the *PeUMPS* cDNA vectors for expression in *P. ellipsoidea*

The pUT1 plasmid (NCBI/DDBJ accession number LC016841) carrying the *PeUMPS* cDNA flanked by the *PeTUBULIN1* promoter and the *PeACTIN1* terminator was constructed in two steps (see Additional file 3) using PrimeSTAR MX DNA polymerase (Takara) as described previously [24]. In the first step, three DNA fragments were amplified separately using PCR. Specifically, a 0.9-kb promoter region of *PeTUBULIN1* of *P. ellipsoidea* strain Obi was amplified using primers Ptub_F (5′-CCAAGCTTTGCATGGCATTCAACAAGGTGAA-3′; a HindIII site is underlined) and Ptub_R_Umps5′ (5′-GAGTTGAAGTTGCCATCTTGAACGACTTCCCTCGACAA-3′; an adapter sequence for the second-step PCR is underlined), and a 1.5-kb terminator region of *PeACTIN1* of *P. ellipsoidea* strain Obi was amplified using the primers Umps3′_Tact_F (5′-GCCACGCTGGCATGAGGTGAGCCTGAAACAGCTTTCTG-3′; an adapter sequence for the second-step PCR is underlined) and Tact_R (5′-TCGGTACCGGTTGTTGAGTTGAGCACTGGAC-3′; a KpnI site is underlined) with pG418-T1A DNA [15] as the template. In parallel, a 1.5-kb *PeUMPS* cDNA was amplified using the primers Ptub3′_Umps_CDRF (5′-GGGAAGTCGTTCAAGATGGCAACTTCAACTCCCTCAG-3′; an adapter sequence for the second-step PCR is underlined) and Umps_CDRR_Tact5′ (5′-TGTTTCAGGCTCACCTCATGCCAGCGTGGCCTCATA-3′, an adapter sequence for the second-step PCR is underlined) and pUC-c*PeUMPS* plasmid as the template. In the second step, the three fragments amplified in the first step were assembled into a single fragment using PCR with the three fragment DNAs as the templates and the primers Ptub_F and Tact_R. The resulting DNA fragment was purified using a PCR purification kit (Qiagen), digested using HindIII and KpnI, and cloned into the HindIII and KpnI sites of pBluescript II sk (+).

Next, the *PeUMPS* cDNA was placed between two DNA fragments: one containing the promoter—5′ untranslated region—first intron region of *PeRBCS* and the other containing the 3′ untranslated region—terminator sequence of *PeRBCS*. Briefly, the 1.5-kb *PeUMPS* cDNA was amplified using PCR with the primers Umps_CDRF and Umps_CDRR with pUC-c*PeUMPS* DNA as the template. The PCR product was digested using NcoI and HindIII, and then cloned into the NcoI and HindIII sites of pHA1011 (NCBI/DDBJ accession number LC016840) between the promoter and terminator regions of *PeRBCS* to construct pUT2 (NCBI/DDBJ accession number LC016842).

### Transformation of *P. ellipsoidea* cells

Nuclear transformation was performed using particle bombardment as described previously [15], with slight modifications. Uracil auxotrophic strains were grown to an OD_750_ of 0.5 in MA5 medium supplemented with 1 mM uracil, and 1 ml of the culture was spread on an MA5 minimal agar plate containing 1 mM uracil. After drying the surface of the plate, samples were incubated at 25 °C for 3 days under 12-h light/dark cycles at a light intensity of 50 μmol m^−2^ s^−1^. Then, the plates were placed under the stopping screen of a Biolistic PDS-1000/He particle delivery system (Bio-Rad) at a distance of 6 cm. DNA-coated gold particles of either 0.6 μm (Bio-Rad) or 0.3 μm (Recenttec) diameter were prepared according to the instructions given by Bio-Rad (technical note # 2453), and the cells on MA5 minimal agar plates were bombarded twice at helium pressures of 1100 or 1550 psi, respectively, *in vacuo* with 125 mm Hg. After bombardment, the cells were incubated at 25 °C in the dark for 24 h, and then transferred onto appropriate selection plates.

For the isolation of G418-resistant transformants, cells were suspended in 1 ml of MA5 minimal medium, spread onto MA5 minimal agar plates containing 100 μg ml^−1^ G418 and 1 mM uracil, and incubated in a CO_2_ incubator at 5 % (*v*/*v*) CO_2_ and 25 °C under fluorescence tubes at 50 μmol photon m^−2^ s^−1^. The numbers of G418-resistant colonies that developed on the selection plates were counted after incubation for 14 days to calculate the transformation frequencies. Each of the single colonies that developed on the agar plates was streaked onto fresh MA5 minimal agar plates containing 100 μg ml^−1^ G418 and 1 mM uracil. PCR templates for genotyping the G418-resistant transformants were prepared from cells grown on the agar plates as follows: the cells were suspended in 50 μl of TE buffer (pH 8.0), disrupted by freezing in liquid nitrogen and thawing at 70 °C, and cell debris was removed by centrifugation. PCR was then performed to identify G418-resistant clones using the supernatant as the template and the primers G418F (5′-GATCGGCCATTGAACAAGAT-3′) and G418R (5′-GCGATACCGTAAAGCACGAG-3′). In addition, PCR to detect *PeUMPS* cDNA was performed using the primers Umps_CDRF and Umps_CDRR. PCR to determine a pUT1 region integrated in the genomes of transgenic strains was performed using either Umps_CDRF or Ptub_F as the forward primer, and the reverse primers Umps_CDRR, Tact_R2 (5′-CCCCAGCTGCTACTGCTATC-3′), or Tact_R as shown in Fig. 2. PCR to determine a pUT2 region integrated in the genomes of transgenic strains was performed using Prbc_F (5′-CTCAATCGGCCGATTTCATGCATGA-3′) or Umps_CDRF as the forward primer, and Umps_CDRR or Trbc_R (5′-GTGCTGGTCTGTTTCCATGCAGTCAT-3′) as the reverse primer, as shown in Fig. 4.

For the direct isolation of Ura^+^ transformants, pUT2 plasmid was introduced into strain M2 using particle bombardment as described above. After bombardment, the cells were spread onto MA5 minimal plates solidified with Noble agar. Each single colony that developed on the Noble agar plates was streaked onto fresh MA5 minimal agar plates, and cells that grew on the plates were used for genotype analyses. The PCR to detect *UMPS* cDNA in Ura^+^ transformants was performed using the primers Umps_CDRF and Umps_CDRR.

### Southern blotting

Three micrograms of genomic DNA was digested using restriction enzyme EcoRI or XbaI, separated on 0.8 % (*w*/*v*) agarose gels, and blotted onto a Hybond-N+ (GE Healthcare, UK) using a standard capillary transfer method with 20× SSC as the transfer buffer. The blotted filter was baked at 80 °C for 2 h, and the *PeUMPS* cDNA fragment was labeled using a DIG High Prime DNA labeling and detection kit (Roche Applied Sciences). Hybridization and signal detection were performed according to the manufacturer’s instructions.

### Real-time PCR

Total RNA was extracted from cells grown in MA5 minimal medium to an OD_750_ of 2.0 using a TRIzol® plus RNA purification kit (Ambion), and remaining DNA was digested using a TURBO DNA-free kit (Ambion) according to the manufacturer’s instructions. First-strand cDNA was synthesized using a PrimeScript™ RT reagent kit with gDNA Eraser (Perfect Real Time, TaKaRa) and an RT primer mix containing oligo (dT)_18_ and random hexamers. Real-time PCR (95 °C for 30 s, followed by 40 cycles of 95 °C for 5 s, and 60 °C for 30 s) to quantify *PeUMPS* cDNA was performed using the primers Umps_QRT_F (5′-TCTTCGACAAGTGGGATGATGG-3′; position 908–930 of the 1597-bp *UMPS* cDNA) and Umps_QRT_R (5′-TGTTACCGATGTCCGCAAAC-3′; position 993–1012 of the 1597-bp *UMPS* cDNA) on a TaKaRa Thermal Cycler Dice® real-time system II using SYBR® Premix Ex Taq™ II (Tli RNaseH Plus, TaKaRa) according to the manufacturer’s instructions. The levels of 18S rRNA were also determined as an internal control using the primers 18SrRNA_QRT_F (5′-GGATCAATTGGAGGGCAAGT-3′; position 627–646 of the 1899-bp 18S rRNA) and 18SrRNA_QRT_R (5′-GCCCGAAATCCAACTACGAG-3′; position 713–732 of the 1899-bp 18S rRNA). The melting curves for each PCR product were determined by measuring the decrease in fluorescence with increasing temperature from 60 to 95 °C. Real-time PCR without a template was also performed in each experiment as a negative control.

### Accession numbers

Sequence data from this study can be found in the DDBJ/NCBI data libraries under the accession numbers LC016838 (*PeUMPS*), LC016839 (*PeUMPS*_cDNA), LC016841 (pUT1), LC016840 (pHA1011), and LC016842 (pUT2).

## Additional files

Additional file 1:
**UV radiation survival curves.** UV radiation survival curves for *P. ellipsoidea* strain Obi. Additional file 2:
**Southern blotting.** (A) The restriction sites in the 13-kb genomic DNA region comprising *PeUMPS* are shown. (B) The restriction sites in pUT1 and pUT2 are shown. (C) Southern blotting of genomic DNAs isolated from eight transgenic strains. Genomic DNA was digested using EcoRI or XbaI, and hybridized with a digoxigenin-labeled *PeUMPS* cDNA. Lane PC, pUT2 plasmid digested with HindIII and XbaI as a positive control; lane 1, M4; lanes 2–5, representative transgenic M4 with pUT1 (M4-1D, M4-1C, M4-1A, and M4-1B); lanes 6–9, representative transgenic M4 with pUT2 (M4-2A, M4-2B, M4-2D, and M4-2E). The numbers on the left and right sides of the gels indicate molecular size expressed in kilobase pairs. Arrowheads indicate restriction fragments containing the chromosomal *UMPS* sequence. Additional file 3:
**Construction of pUT1 in two-step PCR.** Ptub_F and Tact_R carry HindIII and KpnI site, respectively, that were used for the cloning into pBluescript II sk(+).

## Abbreviations

5-FOA: 5-fluoroorotic acid
CDR: coding region
ODCase: orotidine-5′-phosphate decarboxylase
OPRTase: oroate phosphoribosyltransferase
*PeACTIN1*: actin gene
*PeRBCS*: 1,5-bisphosphate carboxylase/oxygenase small subunit gene
*PeTUBULIN1*: beta-tubulin gene
*PeUMPS*: uridine monophosphate synthase gene
TALEN: transcription activator-like effector nuclease
UMPS: uridine monophosphate synthetase
Ura+: uracil synthesis
UV: ultraviolet light
