# Supplementary figures and images for: Construction of a self-cloning system in the unicellular green alga Pseudochoricystis ellipsoidea
Source: Biotechnol Biofuels. 2015 Jun 30;8:94. doi: 10.1186/s13068-015-0277-0 (PMC4489027; doi:10.1186/s13068-015-0277-0)

## Slide 1
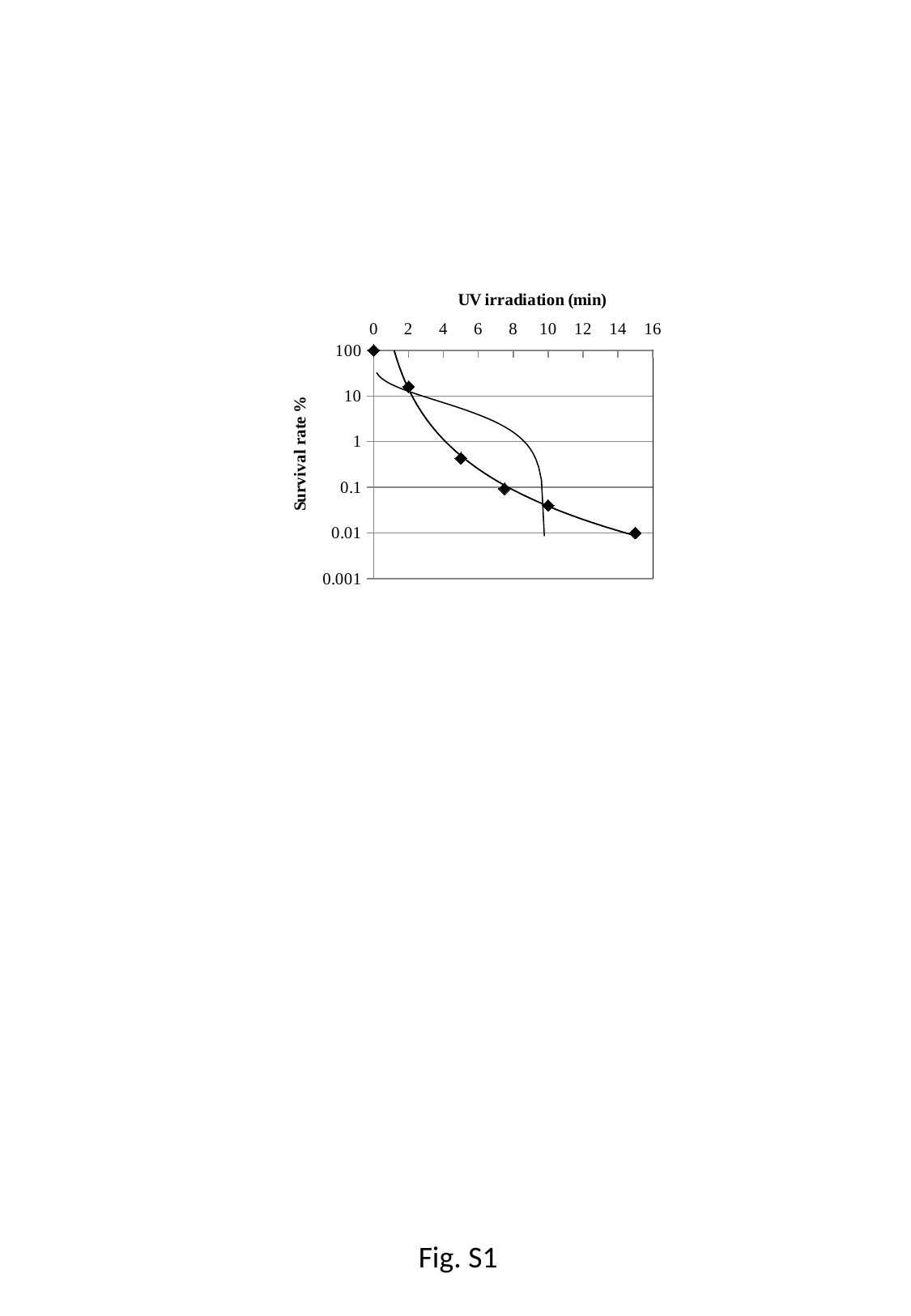

### Chart
| Category | Suvival % |
|---|---|Fig. S1

Supplement: Additional file 1: — UV radiation survival curves. UV radiation survival curves for P. ellipsoidea strain Obi. [file 13068_2015_277_MOESM1_ESM.pptx]
