# Supplementary material for: Construction of a self-cloning system in the unicellular green alga Pseudochoricystis ellipsoidea
Source: Biotechnol Biofuels. 2015 Jun 30;8:94. doi: 10.1186/s13068-015-0277-0 (PMC4489027; doi:10.1186/s13068-015-0277-0)
Supplement: Additional file 2: — Southern blotting. (A) The restriction sites in the 13-kb genomic DNA region comprising PeUMPS are shown. (B) The restriction sites in pUT1 and pUT2 are shown. (C) Southern blotting of genomic DNAs isolated from eight transgenic strains. Genomic DNA was digested using EcoRI or XbaI, and hybridized with a digoxigenin-labeled PeUMPS cDNA. Lane PC, pUT2 plasmid digested with HindIII and XbaI as a positive control; lane 1, M4; lanes 2–5, representative transgenic M4 with pUT1 (M4-1D, M4-1C, M4-1A, and M4-1B); lanes 6–9, representative transgenic M4 with pUT2 (M4-2A, M4-2B, M4-2D, and M4-2E). The numbers on the left and right sides of the gels indicate molecular size expressed in kilobase pairs. Arrowheads indicate restriction fragments containing the chromosomal UMPS sequence. [file 13068_2015_277_MOESM2_ESM.pptx]

## Slide 1
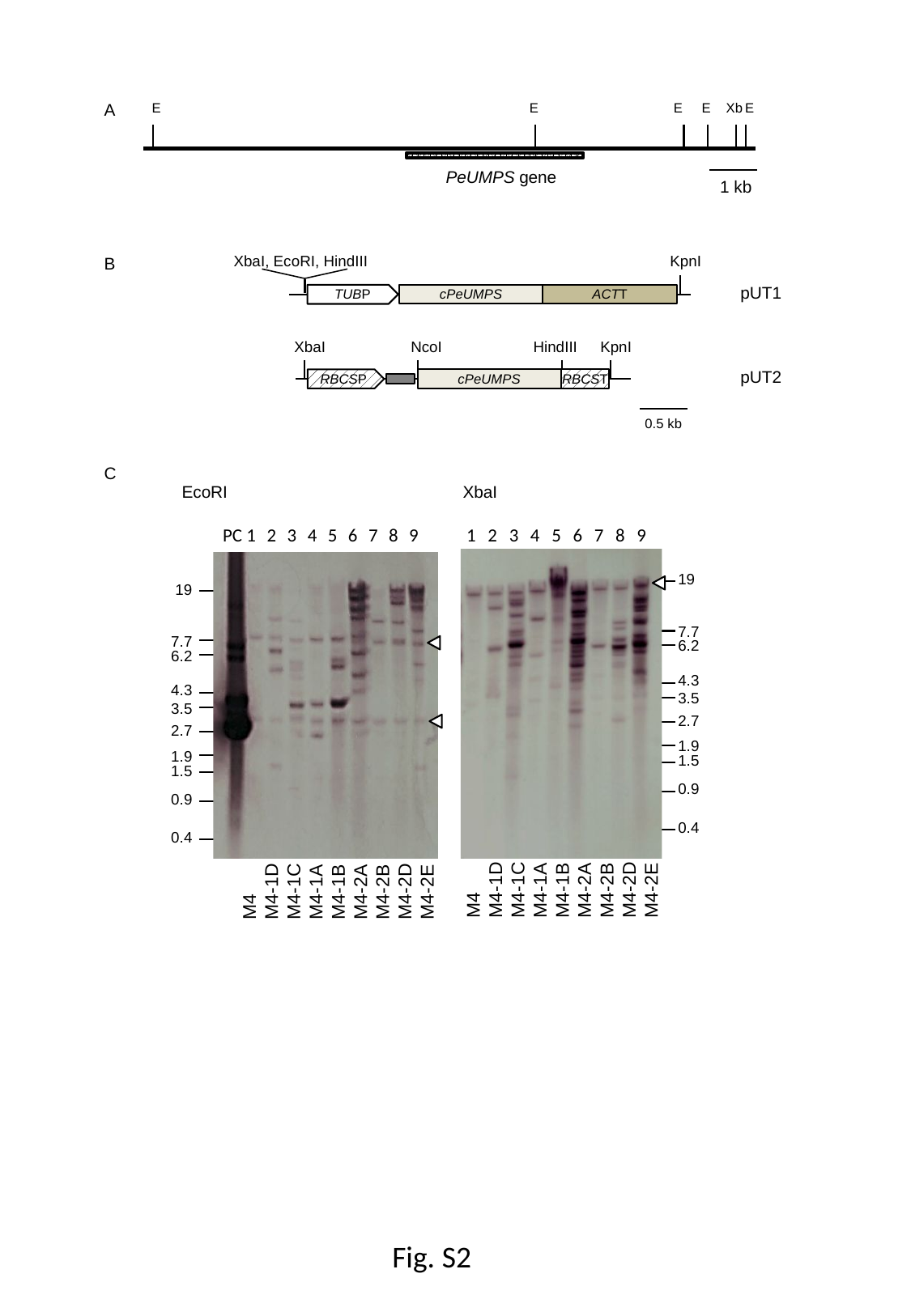

A
E
E
E
E
Xb
E
PeUMPS gene
1 kb
XbaI, EcoRI, HindIII
KpnI
TUBP
cPeUMPS
ACTT
B
pUT1
XbaI
NcoI
HindIII
KpnI
cPeUMPS
RBCST
RBCSP
pUT2
0.5 kb
C
EcoRI
XbaI
PC
1
2
3
4
5
6
7
8
9
19
7.7
6.2
4.3
3.5
2.7
1.9
1.5
0.9
0.4
1
2
3
4
5
6
7
8
9
19
7.7
6.2
4.3
3.5
2.7
1.9
1.5
0.9
0.4
M4
M4-1D
M4-1C
M4-1A
M4-1B
M4-2A
M4-2B
M4-2D
M4-2E
M4
M4-1D
M4-1C
M4-1A
M4-1B
M4-2A
M4-2B
M4-2D
M4-2E
Fig. S2
