# Supplementary material for: Construction of a self-cloning system in the unicellular green alga Pseudochoricystis ellipsoidea
Source: Biotechnol Biofuels. 2015 Jun 30;8:94. doi: 10.1186/s13068-015-0277-0 (PMC4489027; doi:10.1186/s13068-015-0277-0)
Supplement: Additional file 3: — Construction of pUT1 in two-step PCR. Ptub_F and Tact_R carry HindIII and KpnI site, respectively, that were used for the cloning into pBluescript II sk(+). [file 13068_2015_277_MOESM3_ESM.pptx]

## Slide 1
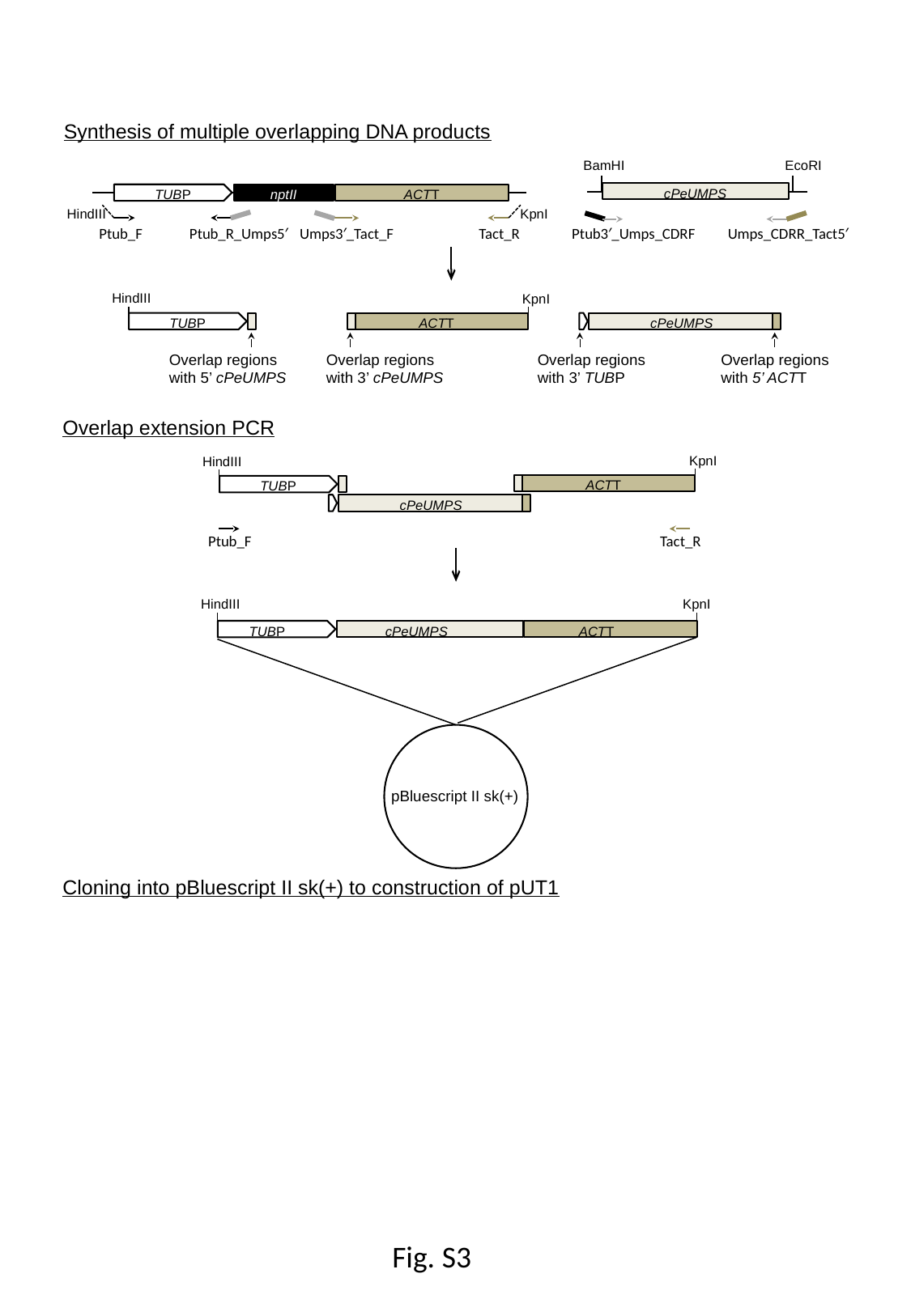

Synthesis of multiple overlapping DNA products
BamHI
EcoRI
cPeUMPS
TUBP
nptII
ACTT
KpnI
HindIII
Ptub_F
Ptub_R_Umps5′
Umps3′_Tact_F
Tact_R
Ptub3′_Umps_CDRF
Umps_CDRR_Tact5′
HindIII
TUBP
KpnI
ACTT
cPeUMPS
Overlap regions with 5’ cPeUMPS
Overlap regions with 3’ cPeUMPS
Overlap regions with 3’ TUBP
Overlap regions with 5’ ACTT
Overlap extension PCR
KpnI
ACTT
HindIII
TUBP
cPeUMPS
Ptub_F
Tact_R
HindIII
KpnI
TUBP
cPeUMPS
ACTT
pBluescript II sk(+)
Cloning into pBluescript II sk(+) to construction of pUT1
Fig. S3
